# Open doors by fair means: Study protocol for a 3-year prospective controlled study with a quasi-experimental design towards (or to implement) an open Ward policy in acute care units

**DOI:** 10.1186/s12888-019-2126-3

**Published:** 2019-05-14

**Authors:** Lisa K. Schreiber, Florian G. Metzger, Tobias A. Duncker, Andreas J. Fallgatter, Tilman Steinert

**Affiliations:** 1 University Department of Psychiatry and Psychotherapy Tuebingen, Calwerstr. 14, 72076 Tuebingen, Germany; 2 Geriatric Center, University Hospital of Tuebingen, Calwerstr. 14, 72076 Tuebingen, Germany; 3 Falkenried Caduceus Klinik, Niendorfer Weg 5, 29549 Bad Bevensen, Germany; 4 Centers for Psychiatry Suedwuerttemberg, Ulm University, Ravensburg-Weissenau, Weingartshofer Str. 2, 88214 Ravensburg, Germany

**Keywords:** Safety, Ward, Psychiatry, Absconding, Coercive measures, Compulsory treatment, Restraint, Absconding, Open door policy, Ward climate, Ward atmosphere, Suicide

## Abstract

**Background:**

Acute psychiatric wards in Germany are often locked due to the assumption that opening could endanger patients and society. On the contrary, some findings suggest that aversive events such as absconding and attempted suicides do not occur more often on wards with an open-door policy. However, these data are probably biased with regard to differing patient populations on open and locked wards. To our best knowledge, the present study is the first prospective controlled study with a quasi-experimental design dealing with this issue.

**Methods:**

This study investigates whether indicators of an open-door policy, as measured by a priori determined outcomes, can be improved by a defined complex intervention on two intervention wards in two psychiatric hospitals, compared to two control wards with otherwise very similar conditions. Both hospitals contain two wards identical in structure and patient admittance policies, so that a similar study protocol can be followed with similar patient populations. Both hospitals have a defined catchment area and receive voluntary and involuntary admissions. In a baseline phase, wards will be opened facultatively (i.e., if it seems possible to staff). In the following intervention period, one ward per hospital will establish an enhanced open-door policy by applying additional strategic and personnel support. As a control group, the control ward will continue to be opened facultatively. After one year, control wards will be opened according to the open-door policy as well. Interventions will include the continuous identification of patients at risk as well as the development of individual care concepts and additional staffing. For this purpose, nursing and medical staff will be methodically supported on an ongoing basis by study staff.

Outcomes variables will be the percentage of door opening on each ward between 8 a.m. and 8 p.m., the percentage of all treatment days with the door opened and the number of involuntary treatment days with open doors. Data on frequencies of aggressive incidents, absconding, police searches, and seclusion or restraint will be used as control variables. Additional costs will be calculated.

**Discussion:**

Treating mentally ill patients on locked wards is a highly relevant and critically discussed topic. In particular, it is controversially discussed whether changes in door policy can be established without increasing risks to patients and others. This study aims to gain robust data on this issue, going beyond beliefs and questionable retrospective observational studies.

**Trial registration:**

Our trial “Open Doors By Fair Means” is retrospectively registered with DRKS (DRKS00015154) on Sept. 10th 2018 and displayed on the public web site. It is searchable via its Meta-registry (http://apps.who.int/trialsearch/).

## Background

In contrast to somatic hospitals, psychiatric clinics are authorized by public and civil law to restrain the patient’s freedom under defined conditions against their will. This involuntary commitment is justified by danger to the patient themselves or others, in Germany as well as in all other developed countries. In the short term this is possible depending on federal state law, e.g. for 24–48 h; in the longer term, the request of the clinic or a legal guardian by judicial authorization is required [1]. Traditionally, involuntary detention is ensured by locking the ward door, in severe cases accompanied by other restrictions such as seclusion or mechanical restraint.

Only about 10% of patients treated in German psychiatric hospitals are held against their will in the clinic [2]. Hence, the treatment of the remaining voluntary 90% of the patients behind locked doors, even if they are allowed to leave the ward at any time, raises ethical questions, such as whether this encroachment on fundamental rights is appropriate and necessary in the treatment of psychiatric patients [3, 4]. Austria has already adopted legislation to address these concerns. Voluntary patients do not need to be treated on locked wards unless they specifically request such conditions (which they usually do not). As a result, in most parts of Austria, psychiatric wards follow an open-door policy which is generally accepted by the public, courts, and the police. In a comparative study on differences between the Weissenau Hospital for Psychiatry in Germany, and the psychiatric hospital in Vienna, Austria, no significant differences were found regarding the frequency of absconding, police searches, suicide attempts, and mechanical restraint within the subgroup of involuntarily treated patients [5].

Since 2015, it is legally possible to treat involuntarily committed patients on open wards in some federal states of Germany [5, 6]. On the background of a movement claiming to abolish all coercive measures in psychiatric hospitals, the practice of locked acute psychiatric care units is a topic of intense discussion in Germany [4, 7]. In order to enable open-ward management, different strategies exist. A relationship-oriented approach with more intensive care for ‘difficult’ patients is preferred. In addition, architectural features such as the location of the nurses’ station, the presence of a counter or at least the presence of a nurse near the door, and the possibility of remote door locking are considered vital. The most extreme strategy is to mechanically restrain or seclude those patients who are at risk of leaving an open ward against medical advice. However, even if this seems to happen in some cases in Austria under conditions of generally open wards and maybe also in German hospitals with a strict open-door policy, most experts would consider such procedure as ethically dubious, if not unlawful [8]. Across Germany, there are considerable differences in hospital organization in terms of specialized wards, concentration or distribution of involuntary patients and so-called “intensive care units” (mostly not available). The most recent guideline of the German Association for Psychiatry and Psychotherapy on the prevention of coercive measures [9] does not express a clear recommendation regarding open wards or hospital organization due to a lack of robust evidence, but generally recommends a policy of least possible restrictions of all kinds. Some German clinics already claim to provide only wards with open doors [10].

Patients admitted or transferred to closed wards usually suffer from severe psychotic or manic symptoms. Others, in a smaller number, are either suicidal in the face of a depressive disorder or show self-harming behaviors. In both cases, restrictive interventions potentially might reinforce existing symptoms such as regressive tendencies or exacerbation of psychotic symptoms due to fear and loss of control [11–13].

50–85% of all suicides occur while patients take approved leave from the psychiatric unit. In most of these cases, patients suffer from affective or substance-related disorders. The concerns about this suicide risk and subsequent judicial liability are a major reason for keeping psychiatric wards locked [11, 14, 15].

However, the suspected risk of absconding seems to increase the probability of absconding: 58% of patients who escape or do not return from a leave name the locked door as one of the reasons [16–19]. Significantly fewer patients absconded or were discharged against medical advice during one year of open ward management in a study in the Department of Psychiatry, University of Basel, Switzerland [20]. Moreover, with the door open, compliance increased and the likelihood of self-harming behaviors, aggression, and refusal of medical treatment decreased.

So far, in contrast to intense discussions among professionals, there is little empirical work on the subject, with mostly retrospective observational data lacking adequate controls. For example, the impact of a change of door policy on coercive measures was investigated over a two-year period in a clinic-wide study in Basel, Switzerland. As a result, no increase in coercive measures on other wards was found and the frequency of coercive measures on the newly opened wards decreased overall [21]. However, there was a redistribution of involuntary patients to the remaining locked wards. In another study, two consecutive periods of six months in one acute care unit, one closed and one mostly open, were compared [22]. While patients did not differ in terms of gender, diagnosis, age and duration of treatment, violent incidents and coercive medication application occurred significantly more frequently during the closed-door period. Moreover, while the ward was open, absconding did not occur more frequently.

In a 15-year observational study, outcomes in 21 German psychiatric hospitals were examined retrospectively [23, 24]. Hospitals were dichotomized into those practicing an “open-ward policy” and those with a “locked-door policy”, defined by expert consensus. The probability of completed suicides was defined as the primary outcome measure for one paper [23], aggression, violence, and coercive interventions for the other [24]. Suicide attempts during treatment and absconding with and without return were defined as secondary outcome measures. In addition, differences between ward types regarding outcomes were examined. As a result, no statistically significant differences were found between hospitals regarding suicides and attempted suicides. Absconding with return occurred more often in hospitals with open-ward policy, whereas absconding without return occurred less frequently. Violence did not differ significantly between hospital types (“open door policy” and “locked door policy”), seclusion and restraint were reported less frequently in hospitals with “open door policy” [24]. All kinds of adverse events occurred less frequent on open wards compared to closed wards. The authors concluded that open wards have a positive effect on reducing aggression.

Although this dataset from 21 German clinics represents the largest database on the issue of an open-door policy and the two papers are often cited by those promoting a strict open-door policy, the study approach was subjected to strong criticism in terms of considerable flaws in design and methods [25]. Data was recorded over a long period of time for other purposes. Calculations were based on a simple division into clinics with a locked or open-door policy which was done retrospectively by the study authors based on personal knowledge. Neither the extent to which doors were actually open over the study period, nor whether they were open or closed during the adverse events nor which other interventions had taken place to avoid suicides, violence, or coercive interventions. Therefore causal interpretations are impossible. Moreover, the finding that violent incidences occur less often on open wards and similar findings relating to suicide attempts and the use of seclusion and restraint might easily be explained by the clinical practice to place patients considered at risk on locked wards, i.e., a serious sample bias. However, psychiatric inpatient treatment is not only about avoiding risks. It also focuses on therapeutic setting and ward atmosphere, which are crucial to its success. These include therapeutic hold as a precondition for therapy commitment, and also the support of the patients among each other [26–28]. To investigate the effects of an open-door policy on ward atmosphere from the nursing staff’s point of view, the parameters “safety”, “therapeutic hold”, and “patient cohesion” as well as the global atmosphere on closed wards, open wards and newly opened wards were measured in a study from a university psychiatric hospital in Switzerland via a questionnaire [23]. The global ward atmosphere was reported to be significantly better on newly opened wards than on closed or open wards. Also, the overall feeling of safety was reported to be above average on newly opened wards compared to closed or open wards. The authors attribute this to the establishment of new procedures for dealing with high-risk patients. This might be a specific measure that goes beyond the effect of door-opening and possibly provides an explanation for the significant difference between newly opened and already open wards. While no significant differences in terms of therapeutic hold were found, patient cohesion at newly opened wards was rated above average. The authors attribute this to the approach of “shared decision making”, which is expected to strengthen patients´ personal responsibility and might lead to a more caring behavior within the patient group. Overall, the authors conclude that it is possible to open closed wards without a higher safety risk and, moreover, that it is suitable for the implementation of a more supportive treatment environment.

Overall, structural conditions and traditions seem to account for the treatment of psychiatric patients behind locked doors. As of today, there are no controlled trials and no comparable populations to underpin the ongoing discussions, which are characterized by ideology and criticism of the current handling of coercive measures.

Therefore, the current study investigates whether an open-door policy can be established and which interventions can be used to care for special patients. We will collect quantitative data on duration of open door intervals, days with open doors and involuntary treatment days, adverse events and additional cost. Moreover, we will collect qualitative data from staff and patients on ward atmosphere, sense of security and general concerns on the open door management as well as qualitative data on the reasons for closing an open ward intermittently. In the course of the ongoing trend towards the opening up of psychiatric hospitals in order to create the least restrictive treatment atmosphere, well-made studies producing reliable numbers are urgently needed. Based on the findings of this study, we want to derive recommendations that can make a substantial contribution to new guidelines. These should facilitate decisions in dealing with difficult patients, to apply the least restrictive measures and provide security with regard to legal uncertainties.

## Methods

This study aims to determine the extent to which a total of four wards in two psychiatric hospitals, where also involuntarily admitted patients are treated, can establish an open-door policy via additional staffing and strategic support. For this purpose, the same intervention is carried out in two psychiatric hospitals in Tuebingen and Friedrichshafen, where the two identical wards are either next to each other (Friedrichshafen), or on superimposed floors (Tuebingen). In both scenarios, one ward will serve as experimental, while the other will serve as control. The patients will be admitted in an alternating fashion. After one year, the ward which had previously been the control ward will receive the intervention. The intervention will be continued at the primary intervention ward. In the control wards, treatment as usual will take place. That is, ward doors will not stay closed throughout the first year but will be opened according to clinical considerations as has been previously practiced (so-called facultatively opened doors). After 12 months, the two control wards will undergo three months of implementation of the new policy and become intervention wards as well. Both hospitals provide similar conditions with identical ward architecture and an alternating patient admittance policy, so that the study can be carried out with the same study protocol and similar patient populations.

### Study design

The present study is a prospective controlled study with a quasi-experimental design.

### Ethics and consent

This study was approved by the ethics committee of Ulm University on March 1st, 2017, No. 313/16 and by the ethics committee of Tuebingen University on June 6th, 2017, No. 170/2017/BO1.

### Study participants

Data of all patients who are admitted to the acute wards of the University Department of Psychiatry and Psychotherapy Tuebingen and the Department for Psychiatry Friedrichshafen (part of the Clinic for Psychiatry and Psychotherapy I of Ulm University), between June 1st 2017 and March 31st 2020 will be collected. There is no specific target age group, but as only adults are treated at the participating wards, all patients will be over the age of 18. The 34 months of data collection include a four-month baseline phase, three months of implementation phase and a total of 27 months of intervention in the intervention wards. The investigation group is defined as those patients treated at the two intervention wards within the first 12-month period. The control group is defined as those patients treated at the other wards without specific interventions during the same period. To control for bias, we will compare distributions of diagnoses and how many patients are admitted involuntarily. In Tuebingen, we will also collect data on how long patients are allowed to leave the ward. In Friedrichshafen, there is no further differentiation, patients are either allowed to leave the ward or they are not. This represents different clinical practices which could not completely be harmonized. Furthermore, the number of patients and staff per day will be recorded.

### Recruitment of participants

Participants will not be actively recruited. All patients who are admitted to one of the two acute wards of the University Department of Psychiatry and Psychotherapy Tuebingen and the Department for Psychiatry Friedrichshafen during the 34-month period, will participate in the study without any exclusion criteria.

### Timeline and procedures

**Fig. 1:**
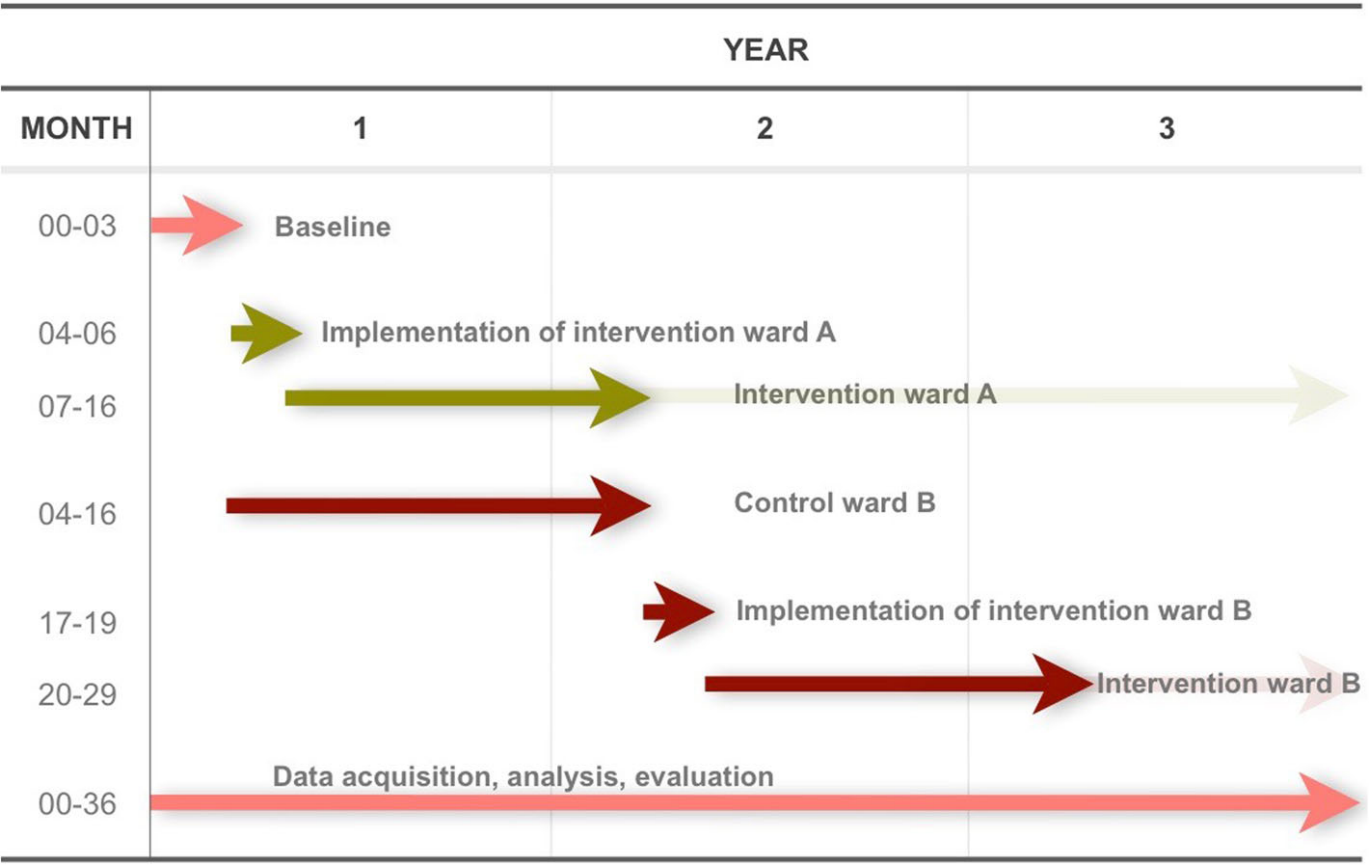
Planned timeline

The planned timeline is shown in Fig. 1.

### Interventions

On each intervention ward, the open-door policy will be implemented as a mission expressed by the clinical director “to keep the door open by all reasonable efforts with high priority, but without running inappropriate risks”. That means it will still be possible to lock the door temporarily with good reasons (to be documented).

Staff will be fully informed about the project and introduced to the interventions (see below) before it starts. Patients will be informed about the open-door policy at admission. As a daily routine, independent of this project, patients come together with nurses to talk about upcoming events each morning. During this meeting, they will be informed about the current door status and questions can be clarified from day to day if necessary.

In detail, interventions will be:

- The door status will be discussed each morning with the complete staffing team (doctors and nurses) and specific reasons requiring a locked door will be identified.
- Weekly team meetings to discuss special events or concerns.
- Patients who are considered to be endangered or at risk, will be identified and the team obliged to discuss possible interventions, e.g. a more intensive care or specific arrangements for patients at risk. These could include accompanied leave, planned visits at home, activities on or outside the wards as well as therapeutic and deescalative talks.
- An additional nurse for the ward team will be deployed in Friedrichshafen beginning with the intervention period; in Tuebingen, nursing trainees will be involved in taking care of patients in need of support.
- An intervention known as the “Potsdam Table” has been implemented successfully several times [28]. This can be a small table with chairs, flowers, newspapers and a nurse as a contact person (professional, nurse) near the ward door. The purpose of this intervention is the establishment of a meeting facility that might dissuade endangered patients urging to leave the ward by deescalating conversations. Depending on the reasons why a patient wishes to leave the ward contrary to the agreement, the contact person can respond by offering contact, initiating activities, discussing the crisis and in case of doubt also deciding that the door should rather be closed.

As doors will only be opened after a careful risk assessment on a daily and situational basis, this approach does not require a termination condition. It will still be possible to close doors at any time when necessary (e.g. if a patient at risk wants to leave and other interventions do not work).

### Standard care

On control wards, the door will continue to be opened facultatively without additional interventions. This requires that all patients accept time agreements, and a sufficient number of staff is always present.

### Outcome measures

The primary outcome measures will be the average opening time of the four acute wards between 8 a.m. and 8 p.m. in percent, the percentage of treatment days with the door open (at least part time), as well as the number of involuntary treatment days with the door open. The latter means that if the door is open for one full day x and seven involuntary patients are present, this will count as 7 involuntary treatment days with open doors.

As secondary outcomes, the frequency of coercive measures (seclusion, restraint, involuntary medication), suicides, suicide attempts, severe self harm and aggressive incidents will be recorded as well as the frequency of absconding. This includes deviations from agreements in voluntarily treated patients, as well as searches for missing patients by the police. In Friedrichshafen, aggressive incidents will be documented with the Staff Observation Aggression Scale [29], in Tuebingen, the documentation of aggressive incidents is part of the survey which is to be filled out on a daily basis. Coercive measures will be routinely documented at both locations by a case register according to the law on assistance for persons with mental diseases in Baden-Wuerttemberg [2]. In addition, costs of the intervention per day with the door open are to be documented. Ward atmosphere will be assessed by staff and patients every six months by the Ward Atmosphere Scale [30].

### Qualitative study

Focus groups will be conducted on each of the four wards with both patients and nurses in different sessions every six months. These group talks contain questions from a semi-structured interview based on the German version of the Ward Atmosphere Scale [30]. Answers and discussions will be audio-taped and evaluated. The acceptance of an open or closed ward management, the ward atmosphere depending on the door status, the feeling of safety and of stigmatization will be addressed. In weekly meetings, the ward team will reflect on problems or uncertainties in the context of the open-door policy during the past week. These meetings will be documented by taking notes. These meetings are thought to help categorize specific difficulties which can be addressed during the planning and implementation of intervention methods.

### Sample size

The size of the sample is equivalent to the total of all patients admitted to the respective hospitals’ acute care units in the intervention period. As an estimate, we expect an amount of 560 admissions per year at each ward in Tuebingen and 420 at each ward in Friedrichshafen. With three months baseline and 24 months of intervention, we expect 4410 patients in 27 months in total.

With regard to the qualitative part of the study, we estimate a total of 280 participants. This includes five times two focus groups with patients and two focus groups with staff separately, with each seven participants for all the four wards, in total 280 patients and 56 professionals.

### Randomization

In this study, no individual randomization is possible because conditions cannot be controlled equally. In the case of two basically identical acute wards, however, cluster randomization is feasible by means of a strictly alternating admission system to one of the two acute wards of the hospital. At baseline, units do not differ in terms of number of admissions, patients´ diagnoses, number or distribution of professional groups. Structural conditions are exactly identical.

### Data collection and management

Data on the door status with registration of reason, time, ID, diagnosis, legal status and explanatory statement will be documented by the nursing staff on a daily basis. Specific interventions for patients at risk during open-ward periods, time and duration, as well as qualitative data from weekly meetings and focus groups will be documented, recorded and transcribed by study staff. Quantitative data on bed occupancy, diagnoses and agreements on leaving the ward will be collected from in-house documentation systems. Data on restraint during survey period will be provided by the department of IT and medical controlling. Staffing levels for each day will be obtained from available nursing schedules.

### Statistical analysis

Calculations will be performed with SPSS.

Descriptive statistics will be obtained. Linear or logistic models for outcomes will be used to examine relationships between explanatory variables and outcomes over time.

For all tests, if necessary, measures will be transformed before analysis. All analyses are exploratory.

### Qualitative data

Thematic analysis will be used for identifying, analyzing and reporting themes or patterns within data. The interviews will be transcribed verbatim and analyzed according to Mayring [31]. This content analysis technique is broadly applied in social sciences to evaluate large quantities of material from semi-structured interviews. It allows to build categories of content and to count certain text components (e.g. aspects of stigmatization, safety feeling, ward atmosphere).

### Collaborating organizations

This study is a collaborative project of the Department of Psychiatry and Psychotherapy of the University Hospital Tuebingen and Department for Psychiatry Friedrichshafen of the Centre for Psychiatry Suedwuerttemberg.

## Discussion

Acute psychiatric care units are often locked in order to prevent the absconding of patients at risk. However, even under the good conditions of hospital treatment in Germany (with more beds available than in many other countries), this classification only applies to about 10% of patients treated in said units. Thus, about 90% of patients are treated on a voluntary basis. Though some of the patients treated voluntarily are at risk of suicide, too, and some of them suffer from serious symptoms, most of them are treated at wards with locked doors without the therapeutic need or legal requirements to do so. Literature offers numerous suggestions on interventions and structural changes in the treatment of acute psychiatric patients. So far, evidence is scarce and focuses on opinions or refers to observational studies.

This study aims to investigate the impact of an open-door policy in a prospective quasi-experimental design on adverse events such as absconding, suicides, suicide attempts, severe self harm and aggressive incidents as well as costs and limitations. More evidence is needed to identify criteria and to develop interventions to modify common treatment methods to a less restrictive, more open and supportive psychiatric care. It is the first prospective controlled study with a quasi-experimental design on this topic.

The results are expected to make a significant contribution to providing sustainable, comprehensive and evidence-based treatment recommendations for acute psychiatric care units and to provide substantial evidence instead of beliefs for a highly controversial discussion.

## References

[1] Meyder J, Wiedwald A, Stolz K, Warmbrunn J, Juchart K (2016). Psychisch-Kranken-Hilfe-Gesetz Baden-Württemberg: Praxiskommentar und Arbeitshilfen.

[2] Flammer E, Steinert T (2018). Das Fallregister für Zwangsmaßnahmen nach dem baden- württembergischen Psychisch-Kranken-Hilfe-Gesetz: Konzeption und erste Auswertungen. Psychiatr Prax.

[3] Steinert T, Lepping P, Bernhardsgrütter R, Conca A, Hatling T, Janssen W, Whittington R (2010). Incidence of seclusion and restraint in psychiatric hospitals: a literature review and survey of international trends. Soc Psychiatry Psychiatr Epidemiol.

[4] Beine KH (2016). Öffnen wir die Türen…. Psychiatr Prax.

[5] Steinert Tilman, Scharfetter Joachim (2018). Wie können psychiatrische Kliniken in Österreich vollständig offen geführt werden?. Psychiatrische Praxis.

[6] Steinert T, Fallgatter A (2016). Psychiatrie mit offenen Türen. Psychiatr Prax.

[7] Zinkler M (2013). Neuregelung von Zwang- ein Auftrag für die Fachgesellschaft?. Psychiatr Prax.

[8] Zinkler M, Nyhuis PW. Offene Türen in der Allgemeinpsychiatrie: Modelle und Standards. Recht Psychiatrie. 2017;35(2).

[9] Fachgesellschaft, H., Steinert, T., Weissenau, Z., & Hirsch, S. S3-Leitlinie „Verhinderung von Zwang: Prävention und Therapie aggressiven Verhaltens bei Erwachsenen “. 10.1007/s00115-019-00801-2 31473766

[10] Noorthoorn E, Lepping P, Janssen W, Hoogendoorn A, Nijman H, Widdershoven G, Steinert T (2015). One-year incidence and prevalence of seclusion: Dutch findings in an international perspective. Soc Psychiatry Psychiatr Epidemiol.

[11] Sollberger D, Lang UE (2014). Psychiatrie mit offenen Türen. Nervenarzt.

[12] Muralidharan S, Fenton M. Containment strategies for people with serious mental illness. Cochrane Database Syst Rev. 2006;(3):CD002084. 10.1002/14651858.CD002084.pub2 PMC11384505 16855984

[13] Combs H, Romm S (2007). Psychiatric inpatient suicide. Primary Psychiatry.

[14] Lang U (2012). Innovative Psychiatrie mit offenen Türen.

[15] Wolfersdorf M, Vogel R, Vogl R, Grebner M, Keller F, Purucker M, Wurst FM (2016). Suizid im psychiatrischen Krankenhaus. Nervenarzt.

[16] Bowers L, Jarrett M, Clark N. Absconding: a literature review. J Psychiatr Ment Health Nurs. 1998;5(5). 10.1046/j.1365-2850.1998.00149.x 10067481

[17] Bowers L, Whittington R, Almvik R, Bergman B, Oud N, Savio M (1999). A European perspective on psychiatric nursing and violent incidents: management, education and service organisation. Int J Nurs Stud.

[18] Falkowski J., Watts V., Falkowski W., Dean T. (1990). Patients Leaving Hospital Without the Knowledge or Permission of Staff–Absconding. British Journal of Psychiatry.

[19] Lang UE, Hartmann S, Schulz-Hartmann S, Gudlowski Y, Ricken R, Munk I, Heinz A (2010). Do locked doors in psychiatric hospitals prevent patients from absconding?. Eur J Psychiatry.

[20] Jungfer HA, Schneeberger AR, Borgwardt S, Walter M, Vogel M, Gairing SK, Huber CG (2014). Reduction of seclusion on a hospital-wide level: successful implementation of a less restrictive policy. J Psychiatr Res.

[21] Blaesi S, Gairing SK, Walter M, Lang UE, Huber CG (2015). Safety, therapeutic hold, and patient's cohesion on closed, recently opened, and open psychiatric wards. Psychiatr Prax.

[22] Baker JA, Bowers L, Owiti JA (2009). Wards features associated with high rates of medication refusal by patients: a large multi-centred survey. Gen Hosp Psychiatry.

[23] Huber CG, Schneeberger AR, Kowalinski E, Fröhlich D, von Felten S, Walter M, Lang UE (2016). Suicide risk and absconding in psychiatric hospitals with and without open door policies: a 15 year, observational study. Lancet Psychiatry.

[24] Schneeberger AR, Kowalinski E, Fröhlich D, Schröder K, von Felten S, Zinkler M, Bux DA (2017). Aggression and violence in psychiatric hospitals with and without open door policies: a 15-year naturalistic observational study. J Psychiatr Res.

[25] Pollmächer T, Steinert T (2016). Arbitrary classification of hospital policy regarding open and locked doors. Lancet Psychiatry.

[26] Steinert T, Bergbauer G, Schmid P, Gebhardt RP (2007). Seclusion and restraint in patients with schizophrenia: clinical and biographical correlates. J Nerv Ment Dis.

[27] Middelboe T, Schjødt T, Byrsting K, Gjerris A (2001). Ward atmosphere in acute psychiatric in- patient care: patients' perceptions, ideals and satisfaction. Acta Psychiatr Scand.

[28] Anschuetz S, Lerch WD, Schulz A (1999). Der Potsdamer Tisch - Therapie- und Sicherheitsfunktion eines Moebelstuecks in einer Psychiatrischen Klinik. Psych. Pflege Heute.

[29] Nijman HL, Muris P, Merckelbach HL, Palmstierna T, Wistedt B, Vos AM, Allertz W (1999). The staff observation aggression scale–revised (SOAS-R). Aggress Behav.

[30] Engel RR, Knab B, von Doblhoff-Thun C (1983). Stationsbeurteilungsbogen (SBB). Diagnostika.

[31] Mayring P. Qualitative content analysis: theoretical foundation, basic procedures and software solution. 2014. http://nbn-resolving.de/urn:nbn:de:0168-ssoar-395173. Accessed 13 Jul 2018.

